# (*E*)-*N*′-(2,5-Dimethoxy­benzyl­idene)-3,4-dihydroxy­benzohydrazide monohydrate

**DOI:** 10.1107/S1600536809039300

**Published:** 2009-10-03

**Authors:** Bin Xu, Ling Han, Qi-Hui Zhang

**Affiliations:** a Department of Nutrition, Jilin Medical College, Jilin 132013, People’s Republic of China; b Liao Ning Benxi Third Pharmaceuticals Co Ltd, Benxi 117004, People’s Republic of China; c College of Traditional Chinese Materia Medica, Shenyang Pharmaceutical University, Shenyang 110016, People’s Republic of China

## Abstract

In the title compound, C_16_H_16_N_2_O_5_·H_2_O, the dihedral angle between the two benzene rings is 25.9 (1)°. Intra­molecular O—H⋯O and N—H⋯O hydrogen bonds are observed. In the crystal, the components are linked into a three-dimensional network by O—H⋯O and O—H⋯(O,O) hydrogen bonds.

## Related literature

For related structures, see: Pu (2008 ▶); Wang *et al.* (2009 ▶). For reference structural data, see: Allen *et al.* (1987 ▶).
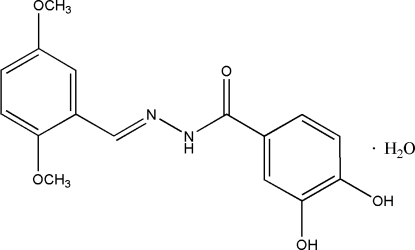


## Experimental

### 

#### Crystal data

C_16_H_16_N_2_O_5_·H_2_O
*M* *_r_* = 334.32
Monoclinic, 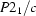

*a* = 10.2573 (8) Å
*b* = 12.4199 (10) Å
*c* = 14.0042 (8) Å
β = 119.666 (4)°
*V* = 1550.2 (2) Å^3^
*Z* = 4
Mo *K*α radiation
μ = 0.11 mm^−1^
*T* = 295 K
0.20 × 0.18 × 0.17 mm

#### Data collection

Siemens SMART CCD diffractometer
Absorption correction: multi-scan (*SADABS*; Siemens, 1996 ▶) *T*_min_ = 0.978, *T*_max_ = 0.981
7993 measured reflections
2735 independent reflections
2110 reflections with *I* > 2σ(*I*)
*R*_int_ = 0.060

#### Refinement

*R*[*F* ^2^ > 2σ(*F* ^2^)] = 0.036
*wR*(*F* ^2^) = 0.098
*S* = 1.02
2735 reflections
220 parameters
H-atom parameters constrained
Δρ_max_ = 0.30 e Å^−3^
Δρ_min_ = −0.15 e Å^−3^

### 

Data collection: *SMART* (Siemens, 1996 ▶); cell refinement: *SAINT* (Siemens, 1996 ▶); data reduction: *SAINT*; program(s) used to solve structure: *SHELXS97* (Sheldrick, 2008 ▶); program(s) used to refine structure: *SHELXL97* (Sheldrick, 2008 ▶); molecular graphics: *SHELXTL* (Sheldrick, 2008 ▶); software used to prepare material for publication: *SHELXTL*.

## Supplementary Material

Crystal structure: contains datablocks global, I. DOI: 10.1107/S1600536809039300/hb5111sup1.cif

Structure factors: contains datablocks I. DOI: 10.1107/S1600536809039300/hb5111Isup2.hkl

Additional supplementary materials:  crystallographic information; 3D view; checkCIF report

## Figures and Tables

**Table 1:** Hydrogen-bond geometry (Å, °)

*D*—H⋯*A*	*D*—H	H⋯*A*	*D*⋯*A*	*D*—H⋯*A*
O1—H1⋯O3^i^	0.82	2.03	2.8384 (15)	168
O2—H2⋯O1	0.82	2.25	2.7014 (15)	115
O2—H2⋯O5^i^	0.82	2.43	3.0191 (15)	130
O6—H17⋯O2^ii^	0.85	2.06	2.9003 (15)	169
O6—H18⋯O3^i^	0.85	1.95	2.7837 (15)	165
N1—H1*A*⋯O6	0.86	2.16	2.8592 (17)	138

Symmetry codes: (i) 
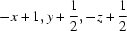
; (ii) 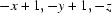.

**Fig. 1.**
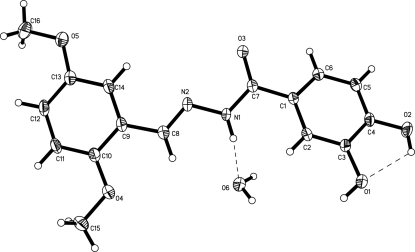
The molecular structure of (I), with displacement ellipsoids drawn at the 30% probability level. The dashed lines indicate hydrogen bonds.

**Fig. 2.**
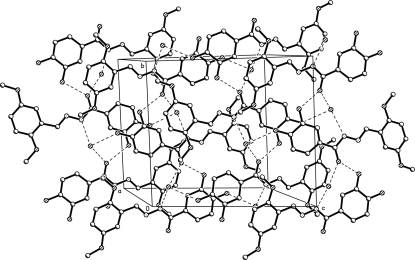
The molecular packing of (I). Intermolecular hydrogen bonds are shown as dashed lines. H atoms not involved in the hydrogen bonds have been omitted for clarity.

## supplementary crystallographic information

### Comment

Schiff base compounds have been of great interest for many years. These compounds play an important role in the development of coordination chemistry related to catalysis and enzymatic reactions, magnetism and molecular architectures (Pu, 2008). As a part of our on going investigation in this field we have determined the crystal structure of the title compound, (I).

The Schiff base molecule of the compound displays a *trans* configuration with respect to the C=N and C—N bonds(Fig. 1). All the bond lengths are within normal ranges (Allen *et al.*, 1987), and are comparable to other Schiff base compounds containing 2,5-dimethoxybenzaldehyde (Wang *et al.*, 2009). The dihedral angle between the two benzene rings is 25.9 (1)°. Intramolecular O—H···O and N—H···O hydrogen bonds are observed (Table 1). Molecules are linked into three-dimensional network by O—H···O hydrogen bonds (Fig. 2).

### Experimental

2,5-Dimethoxybenzaldehyde (0.1 mmol, 16.6 mg) and 3,4-dihydroxybenzohydrazide (0.1 mmol, 16.9 mg) were dissolved in a 95% ethanol solution (10 ml). The mixture was stirred at room temperature to give a clear colorless solution. Light yellow blocks of (I) were formed by gradual evaporation of the solvent over a period of six days at room temperature.

### Refinement

All H atoms were placed in geometrically idealized positions (C—H = 0.93–0.96 Å, O—H = 0.82–0.85 Å and N—H = 0.86 Å) and refined as riding with *U*_iso_(H) = 1.2*U*_eq_(C,*N*) or  1.5*U*_eq_(O).

### Crystal data

**Table:**

C_16_H_16_N_2_O_5_·H_2_O	*F*(000) = 704
*M**_r_* = 334.32	*D*_x_ = 1.432 Mg m^−^^3^
Monoclinic, *P*2_1_/*c*	Mo *K*α radiation, λ = 0.71073 Å
Hall symbol: -P 2ybc	Cell parameters from 2817 reflections
*a* = 10.2573 (8) Å	θ = 2.3–28.0°
*b* = 12.4199 (10) Å	µ = 0.11 mm^−^^1^
*c* = 14.0042 (8) Å	*T* = 295 K
β = 119.666 (4)°	Block, light yellow
*V* = 1550.2 (2) Å^3^	0.20 × 0.18 × 0.17 mm
*Z* = 4	

### Data collection

**Table:**

Siemens SMART CCD diffractometer	2735 independent reflections
Radiation source: fine-focus sealed tube	2110 reflections with *I* > 2σ(*I*)
graphite	*R*_int_ = 0.060
ω scans	θ_max_ = 25.1°, θ_min_ = 2.3°
Absorption correction: multi-scan (*SADABS*; Siemens, 1996)	*h* = −12→10
*T*_min_ = 0.978, *T*_max_ = 0.981	*k* = −14→14
7993 measured reflections	*l* = −16→15

### Refinement

**Table:**

Refinement on *F*^2^	Secondary atom site location: difference Fourier map
Least-squares matrix: full	Hydrogen site location: inferred from neighbouring sites
*R*[*F*^2^ > 2σ(*F*^2^)] = 0.036	H-atom parameters constrained
*wR*(*F*^2^) = 0.098	*w* = 1/[σ^2^(*F*_o_^2^) + (0.0472*P*)^2^ + 0.0836*P*] where *P* = (*F*_o_^2^ + 2*F*_c_^2^)/3
*S* = 1.02	(Δ/σ)_max_ < 0.001
2735 reflections	Δρ_max_ = 0.30 e Å^−^^3^
220 parameters	Δρ_min_ = −0.15 e Å^−^^3^
0 restraints	Extinction correction: *SHELXL97* (Sheldrick, 2008), Fc^*^=kFc[1+0.001xFc^2^λ^3^/sin(2θ)]^-1/4^
Primary atom site location: structure-invariant direct methods	Extinction coefficient: 0.0092 (15)

### Special details

**Table:**

Geometry. All e.s.d.'s (except the e.s.d. in the dihedral angle between two l.s. planes) are estimated using the full covariance matrix. The cell e.s.d.'s are taken into account individually in the estimation of e.s.d.'s in distances, angles and torsion angles; correlations between e.s.d.'s in cell parameters are only used when they are defined by crystal symmetry. An approximate (isotropic) treatment of cell e.s.d.'s is used for estimating e.s.d.'s involving l.s. planes.
Refinement. Refinement of *F*^2^ against ALL reflections. The weighted *R*-factor *wR* and goodness of fit *S* are based on *F*^2^, conventional *R*-factors *R* are based on *F*, with *F* set to zero for negative *F*^2^. The threshold expression of *F*^2^ > σ(*F*^2^) is used only for calculating *R*-factors(gt) *etc*. and is not relevant to the choice of reflections for refinement. *R*-factors based on *F*^2^ are statistically about twice as large as those based on *F*, and *R*- factors based on ALL data will be even larger.

### Fractional atomic coordinates and isotropic or equivalent isotropic displacement parameters (Å^2^)

**Table:**

	*x*	*y*	*z*	*U*_iso_*/*U*_eq_	
O1	0.29807 (12)	0.63600 (8)	0.00386 (8)	0.0415 (3)	
H1	0.3478	0.6763	0.0558	0.062*	
O2	0.25882 (12)	0.49266 (9)	−0.15328 (8)	0.0451 (3)	
H2	0.2479	0.5582	−0.1561	0.068*	
O3	0.50018 (13)	0.24912 (8)	0.30247 (8)	0.0462 (3)	
O4	0.83630 (14)	0.60874 (9)	0.70864 (9)	0.0516 (3)	
O5	0.84768 (13)	0.17847 (9)	0.81326 (9)	0.0554 (4)	
O6	0.71520 (13)	0.60032 (9)	0.33312 (9)	0.0469 (3)	
H17	0.7355	0.5747	0.2857	0.070*	
H18	0.6560	0.6530	0.3021	0.070*	
N1	0.56517 (14)	0.42030 (10)	0.36286 (9)	0.0374 (3)	
H1A	0.5658	0.4873	0.3477	0.045*	
N2	0.62828 (14)	0.38569 (10)	0.47007 (10)	0.0386 (3)	
C1	0.43573 (16)	0.38751 (11)	0.16836 (11)	0.0311 (4)	
C2	0.39529 (16)	0.49534 (12)	0.14219 (11)	0.0324 (4)	
H2A	0.4068	0.5431	0.1970	0.039*	
C3	0.33846 (15)	0.53178 (12)	0.03578 (12)	0.0306 (3)	
C4	0.31715 (16)	0.45885 (12)	−0.04685 (11)	0.0328 (4)	
C5	0.35509 (17)	0.35223 (13)	−0.02211 (12)	0.0380 (4)	
H5	0.3408	0.3041	−0.0773	0.046*	
C6	0.41477 (17)	0.31659 (12)	0.08543 (12)	0.0363 (4)	
H6	0.4409	0.2445	0.1020	0.044*	
C7	0.50232 (16)	0.34597 (12)	0.28220 (12)	0.0336 (4)	
C8	0.69125 (17)	0.45828 (13)	0.54315 (12)	0.0378 (4)	
H8	0.6879	0.5300	0.5230	0.045*	
C9	0.76899 (16)	0.42735 (13)	0.65942 (11)	0.0346 (4)	
C10	0.84706 (17)	0.50414 (13)	0.74194 (12)	0.0364 (4)	
C11	0.92900 (17)	0.46990 (14)	0.84979 (12)	0.0410 (4)	
H11	0.9825	0.5200	0.9049	0.049*	
C12	0.93298 (18)	0.36251 (14)	0.87737 (12)	0.0429 (4)	
H12	0.9899	0.3410	0.9503	0.051*	
C13	0.85242 (18)	0.28700 (13)	0.79661 (12)	0.0396 (4)	
C14	0.77166 (17)	0.32066 (13)	0.68859 (12)	0.0390 (4)	
H14	0.7176	0.2702	0.6340	0.047*	
C15	0.9048 (2)	0.68888 (14)	0.79085 (14)	0.0512 (5)	
H15A	0.8601	0.6884	0.8370	0.077*	
H15B	0.8903	0.7582	0.7567	0.077*	
H15C	1.0102	0.6743	0.8344	0.077*	
C16	0.9447 (2)	0.13853 (17)	0.92118 (15)	0.0683 (6)	
H16A	1.0461	0.1594	0.9442	0.102*	
H16B	0.9382	0.0614	0.9210	0.102*	
H16C	0.9151	0.1678	0.9710	0.102*	

### Atomic displacement parameters (Å^2^)

**Table:**

	*U*^11^	*U*^22^	*U*^33^	*U*^12^	*U*^13^	*U*^23^
O1	0.0508 (7)	0.0360 (6)	0.0266 (6)	0.0042 (5)	0.0106 (5)	0.0021 (5)
O2	0.0597 (8)	0.0478 (7)	0.0227 (6)	0.0049 (5)	0.0166 (6)	0.0044 (5)
O3	0.0635 (8)	0.0327 (6)	0.0257 (6)	−0.0063 (5)	0.0093 (6)	0.0017 (5)
O4	0.0685 (8)	0.0415 (7)	0.0295 (6)	−0.0086 (6)	0.0126 (6)	−0.0060 (5)
O5	0.0657 (8)	0.0467 (7)	0.0324 (7)	−0.0016 (6)	0.0079 (6)	0.0076 (5)
O6	0.0586 (8)	0.0424 (7)	0.0345 (6)	0.0024 (5)	0.0190 (6)	0.0026 (5)
N1	0.0499 (8)	0.0325 (7)	0.0176 (6)	−0.0026 (6)	0.0075 (6)	0.0025 (5)
N2	0.0463 (8)	0.0408 (8)	0.0196 (6)	−0.0029 (6)	0.0093 (6)	0.0009 (6)
C1	0.0319 (8)	0.0328 (8)	0.0222 (7)	−0.0040 (6)	0.0084 (6)	−0.0006 (6)
C2	0.0339 (8)	0.0351 (8)	0.0226 (8)	−0.0032 (6)	0.0097 (7)	−0.0039 (6)
C3	0.0280 (8)	0.0335 (8)	0.0243 (8)	0.0000 (6)	0.0084 (6)	0.0013 (6)
C4	0.0326 (8)	0.0427 (9)	0.0200 (8)	−0.0026 (7)	0.0107 (7)	0.0008 (6)
C5	0.0471 (10)	0.0385 (9)	0.0254 (8)	−0.0036 (7)	0.0156 (7)	−0.0065 (7)
C6	0.0429 (9)	0.0322 (8)	0.0277 (8)	−0.0012 (7)	0.0128 (7)	−0.0007 (7)
C7	0.0361 (9)	0.0337 (9)	0.0238 (8)	−0.0029 (6)	0.0094 (7)	−0.0008 (6)
C8	0.0433 (9)	0.0373 (9)	0.0265 (8)	−0.0001 (7)	0.0125 (7)	−0.0012 (7)
C9	0.0335 (9)	0.0432 (9)	0.0216 (8)	0.0009 (6)	0.0095 (7)	−0.0026 (6)
C10	0.0388 (9)	0.0410 (9)	0.0268 (8)	−0.0007 (7)	0.0143 (7)	−0.0034 (7)
C11	0.0438 (10)	0.0484 (10)	0.0233 (8)	−0.0048 (7)	0.0109 (7)	−0.0091 (7)
C12	0.0454 (10)	0.0542 (11)	0.0204 (8)	0.0024 (8)	0.0097 (7)	0.0005 (7)
C13	0.0425 (10)	0.0427 (10)	0.0278 (8)	0.0002 (7)	0.0130 (7)	0.0009 (7)
C14	0.0413 (9)	0.0433 (10)	0.0240 (8)	−0.0017 (7)	0.0098 (7)	−0.0041 (7)
C15	0.0569 (11)	0.0437 (10)	0.0422 (10)	−0.0060 (8)	0.0162 (9)	−0.0106 (8)
C16	0.0812 (15)	0.0597 (13)	0.0383 (11)	0.0024 (10)	0.0099 (10)	0.0188 (9)

### Geometric parameters (Å, °)

**Table:**

O1—C3	1.3652 (17)	C4—C5	1.375 (2)
O1—H1	0.8200	C5—C6	1.388 (2)
O2—C4	1.3687 (16)	C5—H5	0.9300
O2—H2	0.8198	C6—H6	0.9300
O3—C7	1.2386 (17)	C8—C9	1.466 (2)
O4—C10	1.3662 (19)	C8—H8	0.9300
O4—C15	1.4173 (19)	C9—C14	1.383 (2)
O5—C13	1.3729 (19)	C9—C10	1.404 (2)
O5—C16	1.4254 (19)	C10—C11	1.383 (2)
O6—H17	0.8500	C11—C12	1.384 (2)
O6—H18	0.8508	C11—H11	0.9300
N1—C7	1.3501 (18)	C12—C13	1.384 (2)
N1—N2	1.3770 (17)	C12—H12	0.9300
N1—H1A	0.8595	C13—C14	1.382 (2)
N2—C8	1.2740 (19)	C14—H14	0.9300
C1—C6	1.387 (2)	C15—H15A	0.9600
C1—C2	1.396 (2)	C15—H15B	0.9600
C1—C7	1.482 (2)	C15—H15C	0.9600
C2—C3	1.380 (2)	C16—H16A	0.9600
C2—H2A	0.9300	C16—H16B	0.9600
C3—C4	1.399 (2)	C16—H16C	0.9600
			
C3—O1—H1	109.5	C9—C8—H8	120.4
C4—O2—H2	109.4	C14—C9—C10	119.21 (14)
C10—O4—C15	117.82 (12)	C14—C9—C8	119.97 (14)
C13—O5—C16	117.19 (13)	C10—C9—C8	120.76 (14)
H17—O6—H18	106.2	O4—C10—C11	124.61 (14)
C7—N1—N2	118.09 (12)	O4—C10—C9	116.64 (13)
C7—N1—H1A	120.9	C11—C10—C9	118.75 (15)
N2—N1—H1A	121.0	C10—C11—C12	121.29 (15)
C8—N2—N1	115.64 (13)	C10—C11—H11	119.4
C6—C1—C2	119.12 (13)	C12—C11—H11	119.4
C6—C1—C7	118.53 (13)	C11—C12—C13	120.12 (14)
C2—C1—C7	122.35 (13)	C11—C12—H12	119.9
C3—C2—C1	120.64 (13)	C13—C12—H12	119.9
C3—C2—H2A	119.7	O5—C13—C14	115.51 (14)
C1—C2—H2A	119.7	O5—C13—C12	125.69 (14)
O1—C3—C2	124.19 (13)	C14—C13—C12	118.78 (15)
O1—C3—C4	116.44 (13)	C13—C14—C9	121.79 (15)
C2—C3—C4	119.36 (13)	C13—C14—H14	119.1
O2—C4—C5	119.11 (13)	C9—C14—H14	119.1
O2—C4—C3	120.48 (13)	O4—C15—H15A	109.5
C5—C4—C3	120.41 (13)	O4—C15—H15B	109.5
C4—C5—C6	119.89 (14)	H15A—C15—H15B	109.5
C4—C5—H5	120.1	O4—C15—H15C	109.5
C6—C5—H5	120.1	H15A—C15—H15C	109.5
C1—C6—C5	120.56 (14)	H15B—C15—H15C	109.5
C1—C6—H6	119.7	O5—C16—H16A	109.5
C5—C6—H6	119.7	O5—C16—H16B	109.5
O3—C7—N1	121.84 (13)	H16A—C16—H16B	109.5
O3—C7—C1	122.25 (13)	O5—C16—H16C	109.5
N1—C7—C1	115.91 (12)	H16A—C16—H16C	109.5
N2—C8—C9	119.18 (14)	H16B—C16—H16C	109.5
N2—C8—H8	120.4		
			
C7—N1—N2—C8	177.35 (14)	N1—N2—C8—C9	−176.41 (13)
C6—C1—C2—C3	−1.5 (2)	N2—C8—C9—C14	−0.8 (2)
C7—C1—C2—C3	177.70 (13)	N2—C8—C9—C10	176.25 (15)
C1—C2—C3—O1	−179.19 (13)	C15—O4—C10—C11	−4.8 (2)
C1—C2—C3—C4	2.0 (2)	C15—O4—C10—C9	175.43 (14)
O1—C3—C4—O2	0.1 (2)	C14—C9—C10—O4	−177.86 (14)
C2—C3—C4—O2	179.00 (13)	C8—C9—C10—O4	5.1 (2)
O1—C3—C4—C5	179.83 (13)	C14—C9—C10—C11	2.4 (2)
C2—C3—C4—C5	−1.2 (2)	C8—C9—C10—C11	−174.70 (14)
O2—C4—C5—C6	179.82 (14)	O4—C10—C11—C12	179.15 (15)
C3—C4—C5—C6	0.1 (2)	C9—C10—C11—C12	−1.1 (2)
C2—C1—C6—C5	0.3 (2)	C10—C11—C12—C13	−0.9 (2)
C7—C1—C6—C5	−178.94 (14)	C16—O5—C13—C14	172.04 (16)
C4—C5—C6—C1	0.4 (2)	C16—O5—C13—C12	−6.2 (3)
N2—N1—C7—O3	0.1 (2)	C11—C12—C13—O5	179.70 (15)
N2—N1—C7—C1	179.86 (13)	C11—C12—C13—C14	1.5 (2)
C6—C1—C7—O3	−21.2 (2)	O5—C13—C14—C9	−178.57 (14)
C2—C1—C7—O3	159.56 (15)	C12—C13—C14—C9	−0.2 (2)
C6—C1—C7—N1	159.05 (14)	C10—C9—C14—C13	−1.8 (2)
C2—C1—C7—N1	−20.2 (2)	C8—C9—C14—C13	175.35 (14)

### Hydrogen-bond geometry (Å, °)

**Table:**

*D*—H···*A*	*D*—H	H···*A*	*D*···*A*	*D*—H···*A*
O1—H1···O3^i^	0.82	2.03	2.8384 (15)	168
O2—H2···O1	0.82	2.25	2.7014 (15)	115
O2—H2···O5^i^	0.82	2.43	3.0191 (15)	130
O6—H17···O2^ii^	0.85	2.06	2.9003 (15)	169
O6—H18···O3^i^	0.85	1.95	2.7837 (15)	165
N1—H1A···O6	0.86	2.16	2.8592 (17)	138

Symmetry codes: (i) −*x*+1, *y*+1/2, −*z*+1/2; (ii) −*x*+1, −*y*+1, −*z*.
